# Targeting Anti-Inflammatory Pathways to Treat Diabetes-Induced Neuropathy by 6-Hydroxyflavanone

**DOI:** 10.3390/nu15112552

**Published:** 2023-05-30

**Authors:** Shehla Akbar, Fazal Subhan, Aroosha Akbar, Faiza Habib, Naila Shahbaz, Ashfaq Ahmad, Abdul Wadood, Saad Salman

**Affiliations:** 1 Department of Pharmacy, CECOS University of IT and Emerging Sciences, Peshawar 25000, Pakistan; shehla@cecos.edu.pk (S.A.);; 2 North West Institute of Health Sciences, Peshawar 25000, Pakistan; aroosha.nwihs@gmail.com; 3 Institute of Basic Medical Sciences, Khyber Medical University, Peshawar 25000, Pakistan; faiza.yzai@gmail.com; 4 Department of Pharmacy, Sarhad University of Science and Technology, Peshawar 25000, Pakistan; nailashahbaz55@yahoo.com (N.S.);; 5 Department of Biochemistry, Shankar Abdul Wali Khan University, Mardan 23200, Pakistan

**Keywords:** 6-hydroxyflavanone, diabetes-induced neuropathy, inflammation

## Abstract

It is evident that inflammation and metabolic syndrome instigated by diabetes mellitus can precipitate diabetes-induced neuropathy (DIN) and pain. In order to find an effective therapeutic method for diabetes-related problems, a multi-target-directed ligand model was used. 6-Hydroxyflavanone (6-HF) carrying anti-inflammatory and anti-neuropathic pain potential due to its quadruplicate mechanisms, targeting cyclooxygenase-2 (COX-2), 5-lipoxygenase (5-LOX), and opioid and GABA-A receptors was investigated. The anti-inflammatory potential of the test drug was confirmed utilizing in silico, in vitro, and in vivo tests. A molecular simulation approach was utilized to observe the interaction of 6-HF with the inflammatory enzyme COX-2 as well as opioid and GABA-A receptors. The same was confirmed via in vitro COX-2 and 5-LOX inhibitory assays. In vivo tests were performed to analyze the thermal anti-nociception in the hot-plate analgesiometer and anti-inflammatory action in the carrageenan-induced paw edema model in rodents. The potential anti-nociceptive effect of 6-HF was evaluated in the DIN model in rats. The Naloxone and Pentylenetetrazole (PTZ) antagonists were used to confirm the underlying mechanism of 6-HF. The molecular modeling studies revealed a favorable interaction of 6-HF with the identified protein molecules. In vitro inhibitory studies revealed that 6-HF inhibited the COX-2 and 5-LOX enzymes significantly. The 6-HF at dosages of 15, 30, and 60 mg/kg substantially reduced heat nociception in a hot plate analgesiometer as well as carrageenan-induced paw edema in rodent models. The authors discovered that 6-HF had anti-nociception properties in a streptozotocin-induced diabetic neuropathy model. According to the findings of this study, 6-HF was demonstrated to diminish inflammation caused by diabetes as well as its anti-nociception effect in DIN.

## 1. Introduction

Metabolic syndromes associated with diabetes often lead to critical changes in the body’s homeostasis that manifest as increased oxidative stress and inflammation. Recent studies have shown that inflammation occurs as a result of diabetes-driven hyperlipidemia, culminating in the accumulation of free fatty acids [1]. The chemokines are proinflammatory mediators that are triggered during neuropathic pain. Additionally, metabolic syndrome brought on by diabetes increases the formation of reactive oxygen species (ROS), which in turn triggers a chain reaction that results in the release of additional pro-inflammatory mediators. It is generally known that the nuclear factor (NF), which is the most harmful transcription factor induced by oxidative stress, increases the expression of several genes, most notably the tumor necrosis factor (TNF) and cyclooxygenase-2 (COX-2), once it has been activated [2]. Thus, an uncontrolled blood glucose level facilitates the accumulation of ROS, manifesting as inflammation, which, if not controlled further, gives rise to neuropathy. At present, the treatment strategy involves both peripherally and centrally acting drugs such as TCAs, anti-epileptics, NSAIDS, and opioids [3]; however, the use of these classes of drugs is accompanied by a number of unwanted side effects that usually contribute to a patient’s non-compliance.

**Figure 1:**
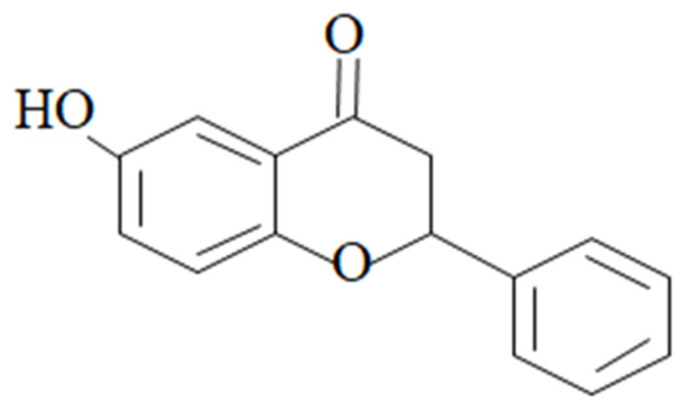
Chemical structure of 6-HF.

Polyphenolic compounds have the potential to display a number of pharmacological properties including anti-inflammatory, anti-oxidant, and anti-neuropathy effects. Previously, we performed various experiments on the flavonoid analogs 6-methoxyflavanone, and we obtained promising results in different neuropathy models [4]. A substitution at position-6 of the flavonoid molecule has been reported to augment the pharmacological potential of the pharmacophore [5]. Consequently, we selected flavonoids belonging to the flavanone family with a substitution at position-6 and different functional groups. We also understood the prominent role of inflammation in instigating chronic pain and the broader picture of the multiple pharmacological activities displayed by flavonoids; this prompted us to examine the multi-target-directed ligand effect of 6-hydroxyflavanone (6-HF) (Figure 1) in treating neuropathic pain via peripheral as well as central mechanisms. The study began with the computational analysis of 6-HF considering the enzymes involved in inflammation, including COX-2, 5-LOX, and the pain-mediating receptors, followed by in vitro inhibitory assays. Finally, an in vivo assessment of the test substance was carried out to verify its efficacy in both an anti-inflammatory model and a model of diabetes-induced neuropathy (DIN).

## 2. Material and Methods

### 2.1. Chemicals and Reagents

All the chemicals/reagents were of analytical grade, including 6-HF, streptozotocin, pentylenetetrazole, celecoxib, and carrageenan, obtained from Sigma-Aldrich, Gillingham, UK; gabapentin, donated by Lowitt Pharmaceuticals, Peshawar, Pakistan; morphine, procured through a proper channel (Punjab Drug House, Lahore, Pakistan); and naloxone, obtained from Hangzhou Uniwise International Corporation, Hangzhou, China.

### 2.2. Animals for Behavioral Studies

The rodents used in this study included female rats (Sprague Dawley, 150–250 g) for neuropathy and carrageenan-induced paw edema tests and mice (BALB/c, 18–30 g) of both sexes in the hot plate analgesiometer retained at an appropriate temperature (22 °C ± 2). These animals were carefully monitored to avoid infections. They received the appropriate nourishment and hydration as needed. The Animal Scientific Procedures Act of 1986 (UK) was strictly adhered to in this study’s experimental procedures, which were approved by the university of Peshawar’s ethical committee (08/EC-15/Pharm).

### 2.3. In Silico Molecular Simulation Studies

Docking studies are a type of computational modeling used to predict the binding of small molecules to target proteins. They are often used in drug discovery and development to identify potential compounds that could bind to a specific target protein and modulate its activity. Flavanones, a class of natural compounds found in many fruits and vegetables, have been the subject of several docking studies to investigate their potential as therapeutic agents [6].

The structures of different opioid receptors were downloaded from PDB (https://www.rcsb.org/, (accessed on 1 September 2022)) with 4DJH (Kappa), 6DDF (MU), 6P3 (Delta), and 6D6T (GABAA) receptors. For the compound, water molecules and co-crystalized ligands were removed, and then the prepared structures were saved as PDB files in Biovia Discovery studio 2021 for further docking steps. The CB-DOCK webserver (http://clab.labshare.cn/cb-dock/php/, (accessed on 2 September 2022)) was used for cavity detection, molecular docking, and to perform docking with its default standard protocol. By using active site prediction tools, the active sites of the receptors for the identification of hydrogen bonds and van der Walls forces were determined [7,8].

### 2.4. In Vitro Cycloxygenase (COX-2) Inhibitory Assay

The COX-2 inhibitory potential of the synthesized compounds was assessed using the following previously reported protocol via a COX colorimetric inhibitor screening assay according to the kit manufacturer’s protocol (Caymanchem, Ann Arbor, MI, USA) [9]. The mixture was maintained at 37 °C for 15 min. Following the completion of the reaction with hydrochloric acid (HCl), the absorbance at 570 nm was determined using a UV-visible spectrophotometer. The absorbance value per unit of time was used to calculate the COX-2 percent inhibition. In this experiment, celecoxib was used as a reference standard. The COX-2 enzyme solution was primed at a 300 U/mL concentration. For the activation, 50 µL of the co-factor solution comprising 1 mM hematin in 0.1 M Tris-buffer at pH 8.0, 0.9 mM glutathione, and 0.24 mM tetramethyl-p-phenylenediamine dihydrochloride (TMPD) was added after 10 µL of the enzyme solution had been maintained on ice (4 °C) for 5 to 6 min. Following that, a test sample (20 µL) and an enzyme solution (60 µL) with varying concentrations were kept at room temperature for 5 to 10 min. Likewise, 30 mM of arachidonic acid (20 µL) was added to initiate the reaction.

### 2.5. In Vitro 5-LOX Inhibition Assay

The inhibitory potential of the different ketone and ketoester derivatives of the succinimides was assessed using human recombinant 5-LOX. Following 10 to 15 min of incubation with the inhibitor at 25 °C, the remaining enzyme potential was used in this test to quantify the degree of enzyme inhibition. The transformation of linoleic acid (the lipoxygenase substrate) into hydroperoxy-octadecadienoate (HPOD) was used to quantify the activity [10].

Ethyl alcohol (Et.OH) was used to dilute linoleic acid to 20 mM. As a result, 1 mL of the enzyme solution (1: 4000) was combined with 100 mL of 2 mM ATP, 100 µL of the inhibitor (1 mM), and 790 µL of Tris buffer before being incubated for 10 min. After mixing the substrate and enzyme for 10 s, another 10 mL of substrate solution (20 mM) was added to the mixture, and the rate of substrate alteration was then observed. As a positive control, the reaction rate without an inhibitor was used. The reference drug in this experiment was nordihydroguaiaretic acid (NDGA).

The activity was measured via the alteration of linoleic acid (lipooxygenase substrate) into hydroperoxy-octadecadienoate (HPOD). The changing rate of absorbance was calculated via a UV-visible spectrophotometer at 234 nm. Tris buffer (50 mM) at pH 7.5, including ethylene diamine tetra acetic acid (EDTA 2 mM) and CaCl_2_ (2 mM), was employed as an assay buffer in this experiment. The enzyme 5-LOX (20,000 U/mL) was diluted to a ratio of 1:4000 with the buffer. A 100 mM quantity of the inhibitor was added to dimethyl sulfoxide (DMSO) and diluted with the assay buffer.

### 2.6. In Vivo Hot Plate Analgesiometer Study

In BALB/c mice weighing 18–22 g, the anti-nociceptive activity of 6-HF was assessed using a hot plate analgesiometer set at 54.0–0.10 °C. After being placed over the hot plate, the animals were observed for reactions such as paw licking, paw flicking, or leaping away from the equipment, which were all deemed responses. To prevent mouse harm, a cut-off period of 30 s was suggested [11].

The mice were pretested 30 min before the medicines were administered in order to evaluate the typical response latencies. Following the pretest, the animals were given diclofenac at a dose of 50 mg/kg; 6-HF at doses of 15, 30, or 60 mg/kg; or morphine (the usual medication) at a dose of 5 mg/kg. Individual mice’s reaction latencies (*n* = 8) were measured 30, 60, and 90 min after injection. Additionally, 1.0 mg/kg of an opioid antagonist (naloxone; injected 10 min before drug administration) and 15 mg/kg of a GABAA receptor antagonist (PTZ; administered 30 min before drug administration) were provided to block the pharmacological activity caused by the medicines.

### 2.7. Carrageenan-Induced Inflammation

A technique for carrageenan-induced inflammation was used to examine 6-HF’s anti-inflammatory effects. In a nutshell, the usual medications, aspirin (50 mg/kg) and 6-HF (15, 30, and 60 mg/kg), were administered to female Sprague Dawley rats (150–200 g; *n* = 6) 30 min before carrageenan (0.05 mL of 1% carrageenan) in the hind paw. Edema (mL) was measured using a digital plethysmometer at various time intervals between 0 and 5 h [12].

### 2.8. Diabetes-Induced Neuropathic Pain (DINP); Development of Diabetes and Neuropathic Pain

For the induction of diabetes mellitus (DM), streptozotocin at a dose of 50 mg/kg was administered to female rats (after fasting for 16 h) with body weights ranging between 150 and 200 g. After 5 days (d) of STZ administration, the animals were subjected to blood glucose evaluation via the tail tip method. The selection criteria for the inclusion of rats in the study protocol were kept as blood glucose >270 mg/dL, and all the rats with blood glucose levels lower than this were excluded from the study [13]. The mechanical allodynia/vulvodynia along with the body weights and blood glucose levels of the selected animals were analyzed at different time points (0, 5,15, and 29 days after STZ injection, respectively). To avoid the occurrence of infections due to polyuria, the sawdust bedding of the animal cages was replaced regularly. The experimental groups were transferred to the behavioral lab and were acclimatized for 30 min on the 29th day post-streptozotocin injection. Following the habituation period, the female rats were administered with the 6-HF or standard gabapentin followed by the assessment of mechanical allodynia or vulvodynia via the up-down method using a von Frey kit [14].

### 2.9. Experimental Protocol

**Figure 2:**
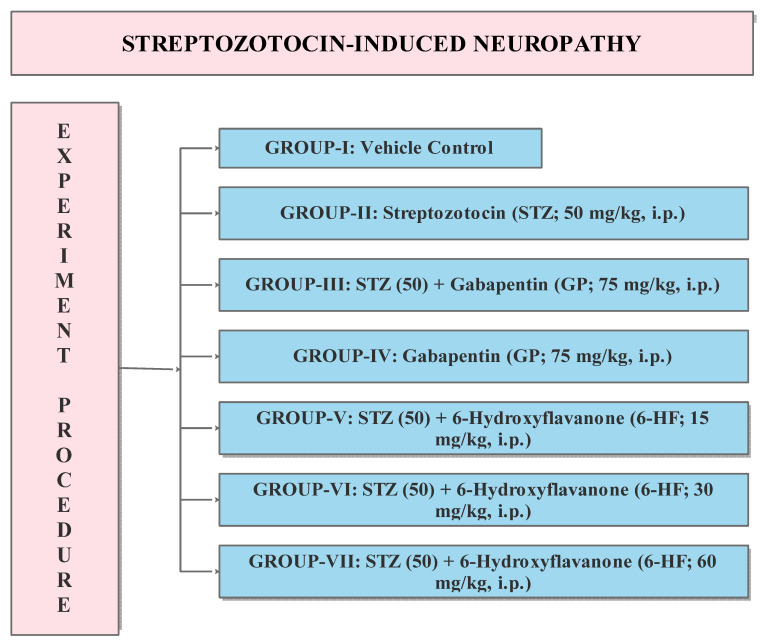
Experimental protocol for the evaluation of 6-HF in DINP model. STZ (50): Streptozotocin 50 mg/kg, GP: Gabapentin 75 mg/kg, 6-HF: 6-hydroxyflavanone at doses of 15, 30, and 60 mg/kg administered intraperitoneally (i.p.) *n* = 8.

The experimental protocol included the division of the animals into seven groups containing 8 rats each, as shown in Figure 2.

### 2.10. Estimation of Static/Dynamic Allodynia and Static/Dynamic Mechanical Vulvodynia

A von Frey filaments kit comprising different forces (0.4, 0.6, 1, 1.4, 2, 4, 6, 8, 10, and 15 g) was used to assess static allodynia. Briefly, the selected force of the filament was subjected vertically to the sole of the right hind paw for a period of 5 s or until the animal lifted its paw. The procedure began with 2.0 g of force, and the point at which the rat lifted its paw was considered as the paw-withdrawal threshold (PWT). A PWT of 3.63 g or below was set as the selection criterion for including animals in the study, along with a cut-off force of 15 g [13]. Similarly, cotton buds gently stroked over the sole region of the rat’s right hind paw were employed to evaluate the dynamic allodynia. The time at which the rat lifted or licked its paw was considered the paw-withdrawal latency (PWL). A PWL of 8 s or below was set as a selection criterion for including animals in the study, along with a cut-off time of 15 s [13].

To evaluate static vulvodynia, we used von Frey filament forces of 0.008, 0.02, 0.04, 0.07, 0.16, 0.4, 0.6, and 1.0 g. The selected force of the filament was subjected vertically to the shaved mucous membrane of the anogenital region for a period of 4 s or until the animal flinched. The procedure began with a 0.04 g force, and the point at which the rat flinched was considered as the flinching-response threshold (FRT). The FRT of 0.16 g or below was set as the selection criterion for including animals in the study, along with a cut-off force of 1.0 g [15]. Consequently, cotton buds gently brushed over the female rats’ mucous membrane in the anogenital region were employed to evaluate the dynamic vulvodynia. The time at which the rat flinched or licked its anogenital area was considered the flinching-response latency (FRL). A FRL of 5 s or below was set as the selection criterion for including animals in the study, along with a cut-off time of 10 s [15].

### 2.11. Statistical Analysis

To calculate the mean, we incorporated data into GraphPad Prism version 5 (Inc., San Diego, CA, USA). Then, one-way ANOVAs were conducted to compare the means between the control and experimental groups. To perform post hoc tests for multiple comparisons, Tukey and Dunnett tests, etc., were performed, and the data were visualized in the form of graphs.

## 3. Results

### 3.1. In Silico: Interaction of 6-HF with the Cyclooxygenase-2 enzymes (COX-2), Opioid and GABA-A Receptors

**Figure 3:**
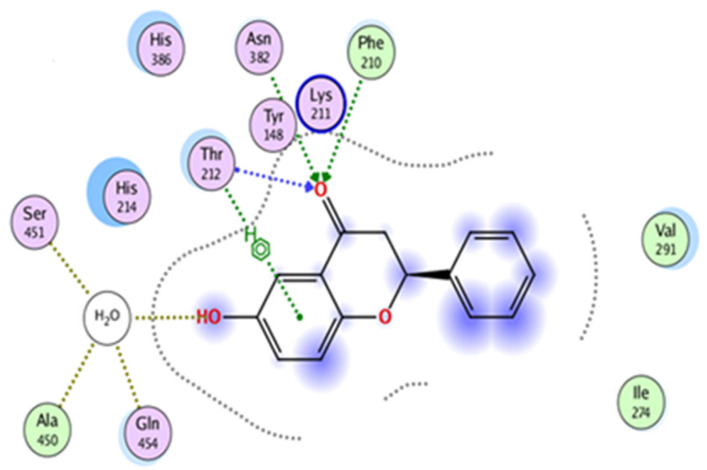
In silico binding of 6-HF with the cyclooxygenase-2 (COX-2) enzyme protein, showing complete interactions with the side chains of the binding sites.

The docking investigations revealed that 6-HF exhibited substantial binding interactions with the residues of the catalytic site. The target enzyme COX-2 (PDB ID: 4m11) was the case, and the 6-HF (docking score = −4.8467) formed three H-bonds with the target enzyme’s active site residues, as shown in Figure 3. The inclusion of the electron-donating group (-OH) may have been the cause of the compound’s effective inhibition.

The docking results also showed that 6-HF had a binding affinity with the delta, kappa, mu-opioid, and GABA_A_ receptors. The 6-HF molecules were capable of making van der Waals and hydrogen bonds (H-bonds) with the corresponding receptors.

**Figure 4:**
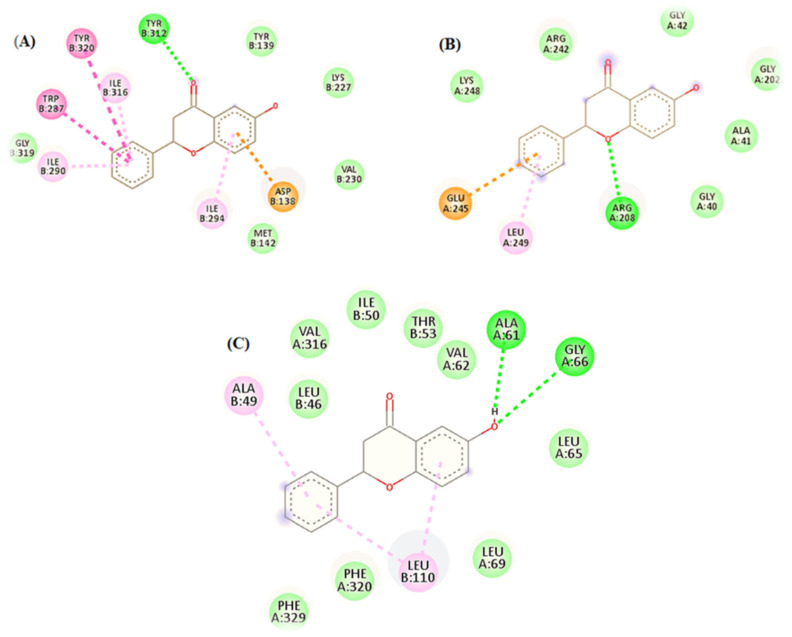
In silico binding of 6-HF with the (**A**) kappa, (**B**) mu, and (**C**) delta opioid receptors, showing complete interactions with the side chains of the binding sites.

The opioid receptors’ ligand-binding regions are situated in an area that is exposed to solvents. The mu-binding receptor has a much bigger opening in its domain than the kappa and delta receptors. Within these pockets, hydrophobic interactions predominately bind morphinan and peptide ligands [16]. Based on the number of interactions with the binding-site residues, the ligand seemed to prefer delta and kappa opioid receptors over mu opioid receptors (Figure 4).

The docking simulations demonstrated two pi–pi interactions between the aromatic side chains of ALA49 and LEU110; two strong H-bonds, ALA61 and GLY66, in the delta opioid receptors (Figure 4C); one strong H-bond interaction, TYRB312; and six pi–pi interactions, including ILE290, ILEB316, ASP138, TYRB320, ILEB294, and TRP287 in the kappa opioid receptors (Figure 4A).

**Figure 5:**
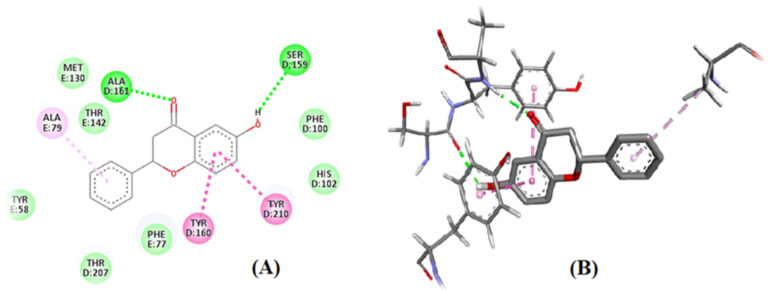
In silico binding of 6-HF with the GABA-A receptors: (**A**) 2D structure, with green color showing the hydrogen bond formation representing the ALA residue (ALA-161) of the α-subunit seen in the 4COF and the hydroxyl group of 6-HF with the side chain of the SER-159 residue in the F-subunit chain of the GABA-A receptor. (**B**) 3D structure, showing interaction of 6-HF with GABA-A receptors.

Two centers of the benzodiazepine binding site were affected by 6-HF. Additionally observed was the pi–pi stacking contact between the benzene ring and the ALA residue (ALA-161) of the subunit of the benzodiazepines. The second binding location was seen toward the other end of the 6-HF bearing the hydroxyl group. It was observed that the hydroxyl group’s electronegative oxygen formed an H-bond with the side chain of the SER-159 residue in the GABAA receptor’s F-subunit chain (Figure 5A,B).

### 3.2. In Vitro Studies: Cycloxygenase (COX-2) and Lipoxygenase (5-LOX) Inhibitory Assay

As shown in Table 1, 6-HF showed significant COX-2 inhibitory activity with an IC50 of 21.86 µM as compared to the standard celecoxib. Similarly, 6-HF also showed significant 5-LOX inhibitory activity with an IC50 of 36 µM as compared to the standard NDGA (Table 2). The percent inhibitory action of 6-HF was dose-dependent.

### 3.3. In Vivo Studies: Evaluation of 6-HF for Thermal Anti-Nociception in the Hot Plate Analgesiometer

**Figure 6:**
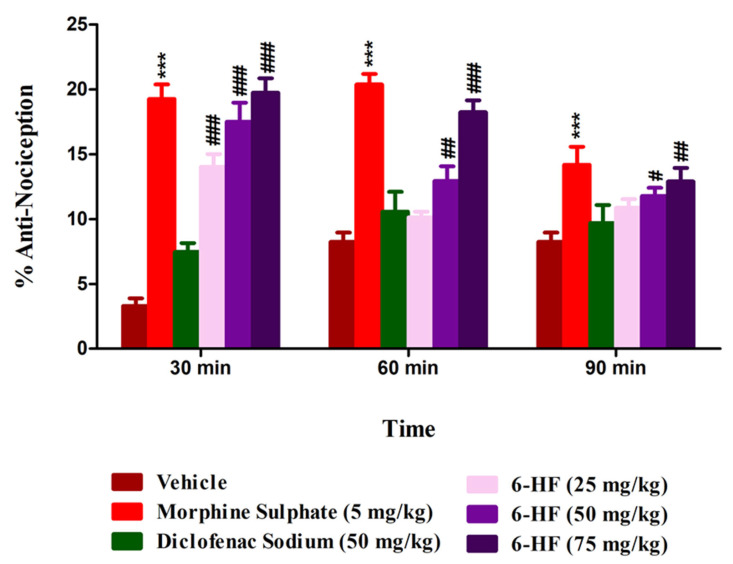
Anti-nociceptive activity of 6-HF at 15, 30, and 60 mg/kg in the hot plate test. *** *p* < 0.001 (MP-5) and ^#^ *p* < 0.05, ^##^ *p* < 0.01, and ^###^ *p* < 0.001 (6-HF) compared to vehicle control animals (ANOVA followed by Dunnett’s post hoc test, *n* = 8).

The 6-HF significantly enhanced the response latencies of the mice treated at doses of 15 mg/kg (*p* < 0.001), 30 mg/kg (*p* < 0.001), and 60 mg/kg (*p* < 0.001) when tested 30 min (F(5,42) = 42.75, *p* < 0.0001) after drug administration in the hot plate analgesiometer. The effect of the higher doses of 6-HF, i.e., 30 mg/kg (*p* < 0.01) and 60 mg/kg (*p* < 0.001), prevailed for 60 min (F(5,42) = 24.24, *p* < 0.0001). The standard morphine showed a significant anti-nociceptive effect at the dose of 5 mg/kg (*p* < 0.001), and this effect prevailed up to 90 min (Figure 6).

**Figure 7:**
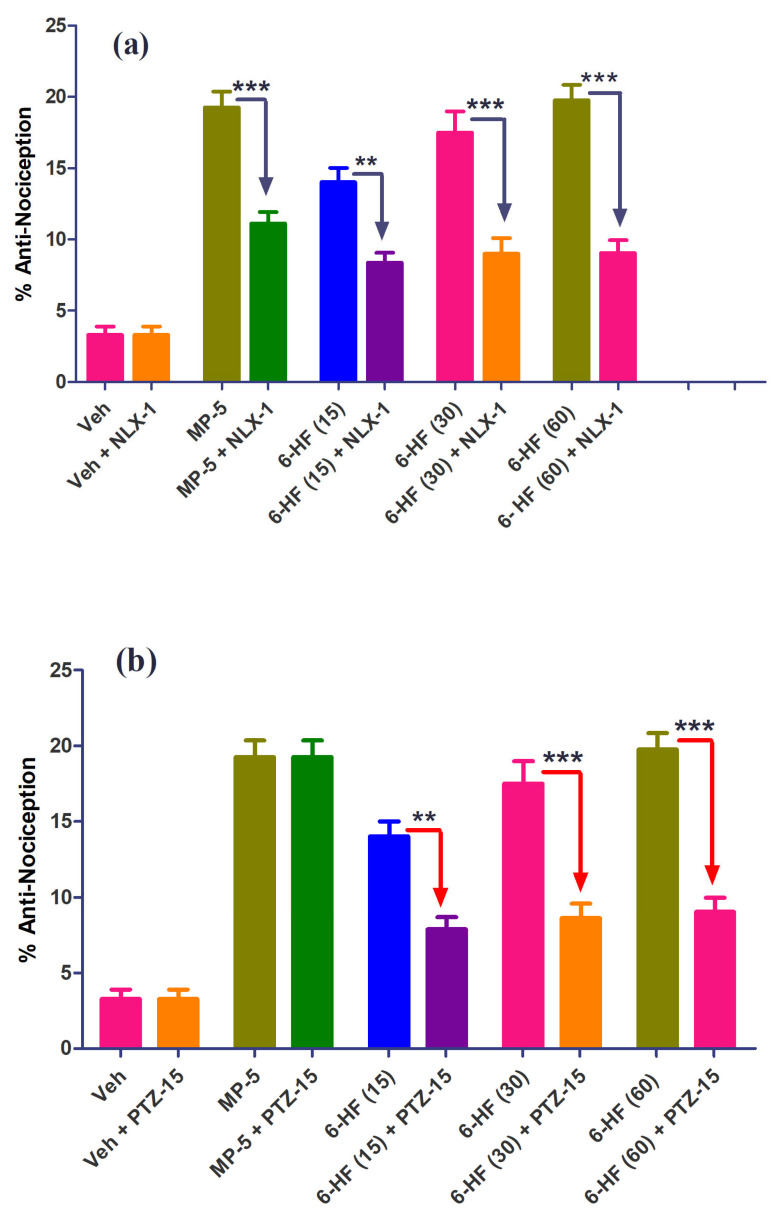
Effect of (**a**) naloxone (1.0 mg/kg; NXL-1) and (**b**) pentylenetetrazole (10 mg/kg; PTZ-10) on the anti-nociceptive effect of 6-HF in the mouse hot plate test. ** *p* ˂ 0.01, *** *p* ˂ 0.001 compared to morphine (5.0 mg/kg; MP-5) or 6-HF (15, 30, and 60 mg/kg) (ANOVA followed by Tukey’s multiple comparison post hoc test, *n* = 8).

The percent anti-nociception induced by the selected doses of 6-HF was significantly antagonized by the concurrent administration of naloxone or PTZ. The co-administration of naloxone (1 mg/kg) (F(9,70) = 38.41; *p* < 0.0001) antagonized the anti-nociception induced by 6-HF at the selected doses of 15, 30, and 60 mg/kg (*p* < 0.001) (Figure 7a). The anti-nociception induced by morphine at the dose of 5 mg/kg was also significantly (*p <* 0.001) antagonized by naloxone. However, the co-administration of PTZ (15 mg/kg) (F(9,70) = 43.27; *p* < 0.0001) with the test drugs only significantly antagonized the anti-nociception induced by 6-HF at the selected doses of 15 (*p* < 0.01), 30 (*p* < 0.001), and 60 mg/kg (*p* < 0.001), while not changing the effects of morphine (Figure 7b).

### 3.4. Anti-Inflammatory Activity of 6-HF

**Figure 8:**
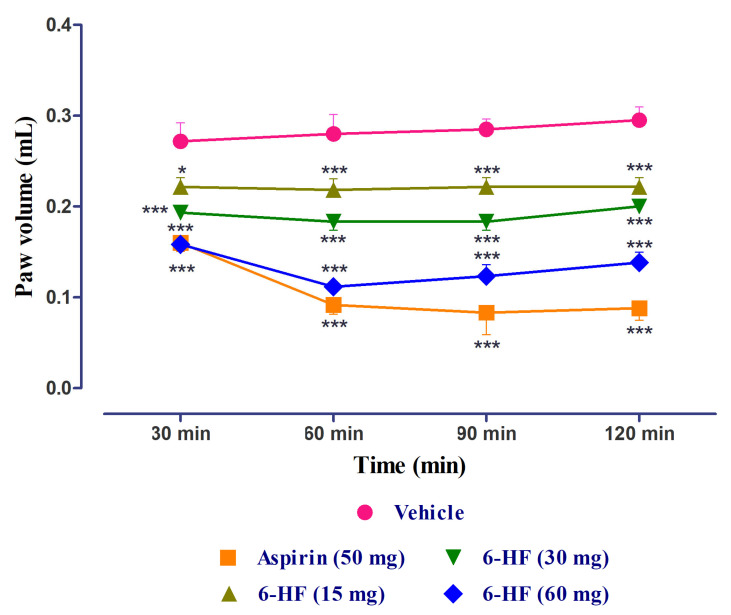
Anti-inflammatory activity of 6-HF at 15, 30, and 60 mg/kg in the carrageenan-induced paw edema test. * p < 0.05, and *** *p* < 0.001 compared to vehicle control animals. Two-way repeated-measure ANOVA post hoc Bonferroni analysis (*n* = 8).

The standard aspirin and 6-HF significantly reduced edema induced by carrageenan (time = (F(3,140) = 4.70, *p* = 0.0037); treatment = (F(4,140) = 199.72, *p* < 0.0001); interaction = (F(12,140) = 4.20, *p* < 0.0001)) when compared to the vehicle control group. The 6-HF was able to reduce the paw volume at all the tested doses of 15, 30, and 60 mg/kg at the time points of 30, 60, 90, and 120 min (Figure 8).

### 3.5. Development of Diabetes Mellitus (DM) by Streptozotocin

**Figure 9:**
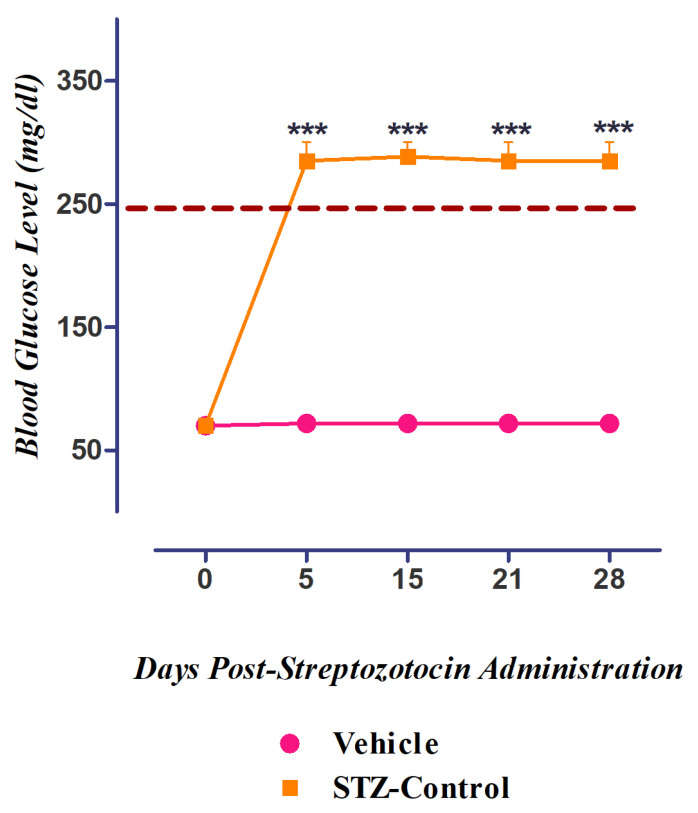
Induction of diabetes in streptozotocin-induced diabetic neuropathic pain model. *** *p* ˂ 0.001 compared to control rats (ANOVA followed by Dunnett’s post hoc test, *n* = 8).

**Figure 10:**
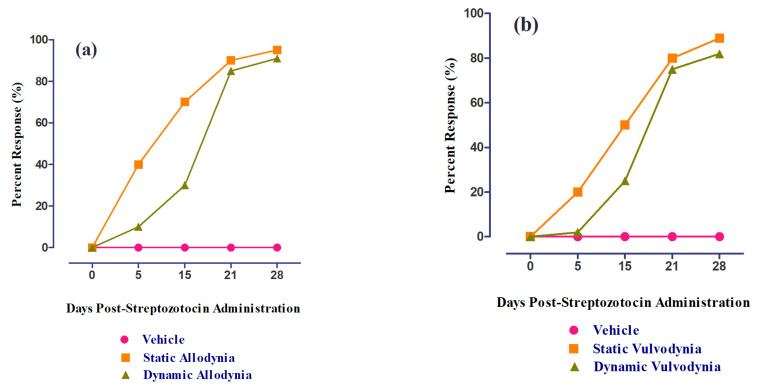
The percent response of female rats post-administration (0, 5,15,21, and 28 d) of streptozotocin: (**a**) static/dynamic allodynia and (**b**) static/dynamic vulvodynia.

DM was successfully developed via the single-dose administration of streptozotocin (50 mg/kg), depicted by the increased blood glucose levels measured at different periods (0, 5 (*p <* 0.001), 15 (*p <* 0.001), and 28 (*p <* 0.001) d post-administration) using a glucometer, as compared to vehicle-treated groups (Figure 9). The percent response in static and dynamic allodynia showed a significant reduction in PWT and PWL 5, 15, and 28 d post-administration (Figure 10a). The percent response in static and dynamic vulvodynia also showed a significant reduction in FRT and FRL 5, 15, and 28 d post-administration (Figure 10b).

### 3.6. Role of 6-HF in Ameliorating Static/Dynamic Allodynia

All the animals included in the study developed mechanical static/dynamic allodynia by the 29th day post-administration of STZ (50 mg/kg), accompanied by a significant decrease (*p* < 0.001) in PWT and PWL when compared to the control group.

**Figure 11:**
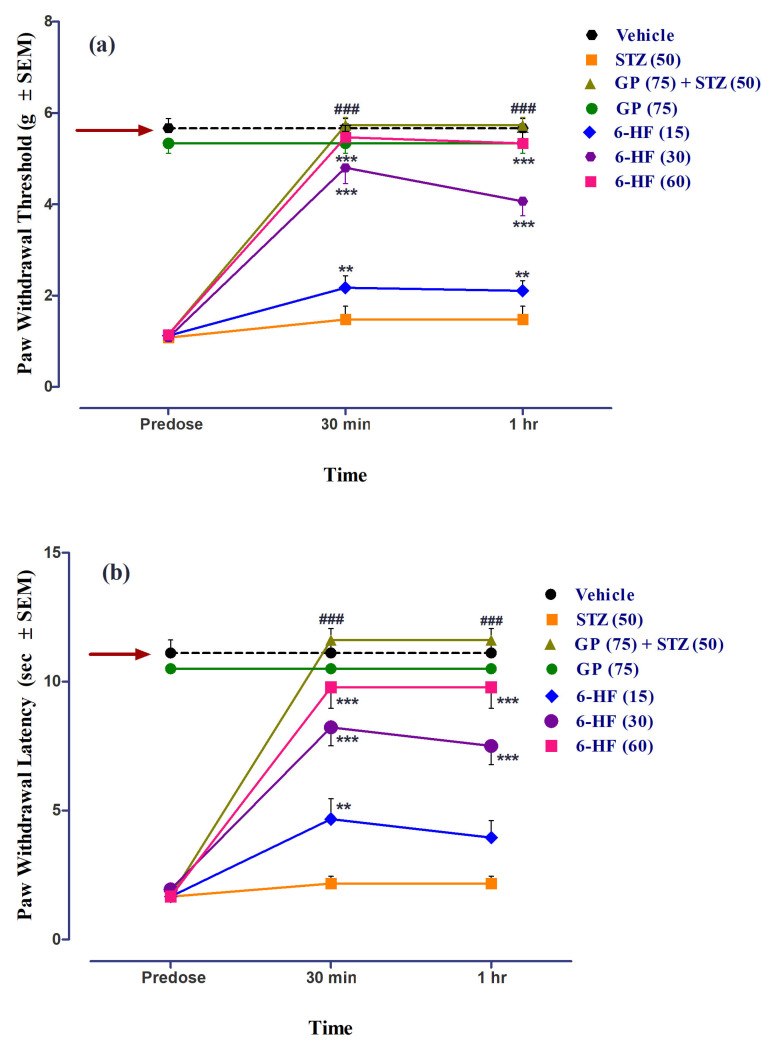
Effect of treatment with 6-HF at doses of 15 mg/kg (6-HF-15), 30 mg/kg (6-HF-30), and 60 mg/kg (6-HF-60) and positive control gabapentin (GP) at a dose of 75 mg/kg (GP-75) on the expression of diabetes-induced mechanical static allodynia (decrease in paw-withdrawal threshold; PWT in g) and dynamic allodynia (decrease in paw-withdrawal latency; PWL in s) in the hind paw of rats. (**a**) Effect of 6-HF and GP on the expression of diabetes-induced static allodynia. (**b**) Effect of 6-HF and GP on the expression of diabetes-induced dynamic allodynia. ^###^ *p* < 0.001 (GP-75) and ** *p* < 0.01, and *** *p* < 0.001 compared to streptozotocin-treated control animals. Two-way repeated-measure ANOVA post hoc Bonferroni analysis (*n* = 8).

6-HF abated STZ-induced mechanical static allodynia (time = (F(2,105) = 193.55, *p <* 0.0001); treatment = (F(6,105) = 176.63, *p* < 0.0001); interaction = (F(12,105) = 30.92, *p <* 0.0001)), as ascertained by the significant increase in the paw-withdrawal threshold (PWT, F(6,35) = 48.68, *p* < 0.0001) at all the doses (15 (*p* < 0.01), 30 (*p* < 0.001), and 60 mg/kg (*p <* 0.001)) 30 min post-administration (Figure 11a).

6-HF was found to reduce mechanical dynamic allodynia (time = (F(2,105) = 130.09, *p* < 0.0001); treatment = (F(6,105) = 129.34, *p* < 0.0001); interaction = (F(12,105) = 21.10, *p <* 0.0001)). Additionally, we ascertained a significant increase in the PWL (F(6,35) = 40.09, *p* < 0.0001) at all doses (15 (*p* < 0.01), 30 (*p* < 0.001), and 60 mg/kg (*p* < 0.001)) 30 min post-administration (Figure 11b).

Gabapentin (75 mg/kg, positive control) significantly increased the paw-withdrawal threshold (PWT, F(6,35) = 48.68, *p* < 0.0001) as well as the paw-withdrawal latency (PWL, F(6,35) = 40.09, *p* < 0.0001). The group of animals receiving only gabapentin did not develop static/dynamic allodynia (Figure 11).

### 3.7. Role of 6-HF in Ameliorating Static/Dynamic Vulvodynia

All the animals included in the study developed mechanical static/dynamic vulvodynia by the 29th day post-administration of STZ (50 mg/kg), accompanied by a significant decrease (*p* < 0.001) in FRT and FRL when compared to the control group.

**Figure 12:**
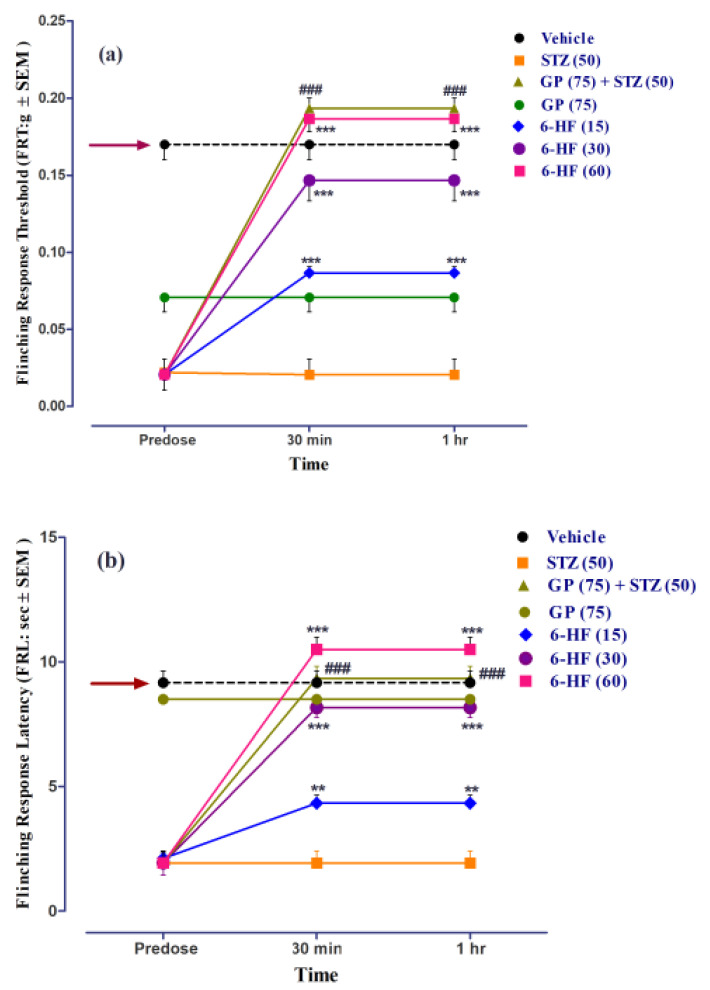
Effect of treatment with 6-HF (6-HF) at doses of 15 mg/kg (6-HF-15), 30 mg/kg (6-HF-30), and 60 mg/kg (6-HF-60) and positive control gabapentin (GP) at a dose of 75 mg/kg (GP-75) on the expression of diabetes-induced mechanical static vulvodynia (decrease in the flinching-response threshold; FRT in g) and dynamic vulvodynia (decrease in the flinching-response latency; FRL in s) in the vulva of rats. (**a**) Effect of 6-HF and GP on the expression of diabetes-induced static vulvodynia. (**b**) Effect of 6-HF and GP on the expression of diabetes-induced dynamic vulvodynia. ^###^ *p* < 0.001 (GP-75) and ** *p* < 0.01, and *** *p* < 0.001 compared to streptozotocin-treated control animals. Two-way repeated-measure ANOVA post hoc Bonferroni analysis (*n* = 8).

6-HF abated STZ-induced mechanical static vulvodynia (time = (F(2,105) = 155.75, *p* < 0.0001); treatment = (F(6,105) = 90.56, *p* < 0.0001); interaction = (F(12,105) = 24.37, *p <* 0.0001)), as ascertained by the significant increase in the flinching-response threshold (FRT, F(6,35) = 51.02, *p* < 0.0001) at all the doses (15 (*p* < 0.001), 30 (*p* < 0.001), and 60 mg/kg (*p* < 0.001)) 30 min and 1 h post-administration (Figure 12a).

6-HF was found to reduce mechanical dynamic vulvodynia (time = (F(2,105) = 128.71, *p* < 0.0001); treatment = (F(6,105) = 94.29, *p* < 0.0001); interaction = (F(12,105) = 21.84, *p <* 0.0001)) Additionally, we ascertained a significant increase in the flinching-response latency (FRL, F(6,35) = 43.47, *p* < 0.0001) at all doses (15 (*p* < 0.01), 30 (*p* < 0.001), and 60 mg/kg (*p* < 0.001)) 30 min post-administration (Figure 12b).

Thegabapentin (75 mg/kg, positive control) significantly increased the flinching-response threshold (FRT, F(6,35) = 51.02, *p* < 0.0001), as well as the flinching-response latency (FRL, F(6,35) = 43.47, *p* < 0.0001). The group of animals receiving only gabapentin did not develop static/dynamic vulvodynia (Figure 12).

## 4. Discussion

Although much research is under way on the potential pharmacological effectiveness of flavonoids in neuropathy models, only a few studies have discussed the underlying mechanisms. The present study focused on the multi-target-directed ligand model to examine 6-HF’s anti-nociceptive and anti-inflammatory potential for treating DINP. Previously, we reported flavanone analogs in neuropathy models and targeted the GABAergic and opioidergic mechanisms for the treatment of neuropathy. However, in this research, the additional mechanism of action of flavanone was explored and successfully related to its anti-nociceptive potential. To achieve this, in vitro testing followed by in vivo confirmatory tests were performed to observe the role of 6-HF in reducing inflammation induced by diabetes.

It is now believed that DM-induced metabolic disorders instigate hyperglycemia, dyslipidemia, and insulin resistance, which activates inflammatory pathways [17]. Hence, inflammation presents as a major co-morbidity associated with DM. This introduces a set of complications that negatively affect the patient’s quality of life, neuropathy being the most prominent. The culprit behind the exaggerated inflammatory responses tends to be oxidative stress induced by poor glycemic control. The oxidative stress activates the CNS, causing an activation of the microglia and initiating a cascade of events culminating in the release of multiple inflammatory mediators. It starts with the initial release of pro-inflammatory cytokines, interleukins, and chemokines from the glial cells and neurons. These pro-inflammatory mediators activate inflammatory pathways and recruit additional inflammatory cells to the area to further exacerbate the inflammatory response. They further worsen the condition by activating the endothelial cells and transcription factors. The endothelial activation leads to the leakiness of the cells and the insertion of the inflammatory cells into the brain and spinal cord. The transcription factors controlling gene expression such as nuclear factor-kappa B (NF-κβ), activator protein-1 (AP-1), and STAT-1 add to the ongoing set of events [18]. Inducible nitric oxide synthase (iNOS) and COX-2 released after microglia activation and gene induction augment the inflammation, thus worsening the symptoms of neuropathy [19,20]. It has been reported that in diabetes, the spinal cord level of COX-2 is significantly increased, causing peripheral neuronal sensitization [17]. It is well-known that Carrageenan administration induces acute inflammation by activating the COX-2 enzyme-mediated inflammatory pathways via IL-6 activation at both the peripheral and spinal cord levels, within 2–4 h post-administration [21]. COX-2 expression by carrageenan is related to the induction of hyperalgesia, which was significantly inhibited by intrathecal or systematic COX-2 inhibitors. COX-2 inhibition is associated with the suppression of IL-1β-induced substance-P from the primary afferent neurons [22]. The drug molecules effective in blocking both COX and 5-LOX enzymes are considered as an ideal therapy for treating inflammatory disorders [9]. Hence, 6-HF demonstrated an anti-inflammatory effect by inhibiting both COX-2 and 5-LOX enzymes, as confirmed by both in silico and in vitro inhibitory assays.

The promising role of flavonoids in inflammation is well-documented, the pertinent target being the NF-κβ and MAPK-induced cascade of events resulting in exaggerated inflammatory responses in the neurons [23]. Citrus flavonoids such as hesperidine, diosmin, isohoifolin, naringin, neohesperidine, neoeriocitrin, diosmetin, luteolin, and kaempferol are reported to have a significant effect on the inflammatory markers involved in inflammation. In this regard, Kiruthiga et al. reported the COX-2 inhibitory activity of flavonone via computational in vitro and cell-based assays [24]. Similarly, cell-based studies on kaempferol showed a significant inhibition of IL-6 induced COX-2 expression [25]. Flavonoids also possess anti-oxidant activity; however, the structure–activity relationships show that the presence of two to three double bonds and a substitution at the position 3 are essential for the antioxidant activity of the group [26]. We also performed DPPH free-radical scavenging tests for 6-HF, but the results were not significant, thus aligning with the previous research, as flavanone molecules lack two to three double bond.

6-HF greatly reduced vulvodynia and allodynia precipitated by diabetes. Peripheral neuropathy alters the electrical characteristics of sensory nerves, resulting in an imbalance between central excitatory and inhibitory transmission, which damages inhibitory interneurons and descending regulatory networks. As a result, the dis-inhibition or facilitation mechanisms impede sensory signal transmission in the spinal dorsal horn neurons. Numerous preliminary investigations have shown that neuropathic pain causes an increase in excitation and a decrease in inhibition at the periphery, which leads to hyperexcitability.

The continuation of these sequences may result in the development of persistent neuropathic pain. GABAergic neurons make up 20 to 30% of the CNS’s neurons and are responsible for controlling emotions, pain, muscle tone, and cognition [27]. Therefore, the CNS experiences a variety of unpleasant states as a result of the lack of GABA-mediated inhibition.

It is well-known that GABA_A_ receptors regulate neuropathic pain [28,29,30]. In this regard, it has been reported that the GABA_A_ receptor agonists muscimol and isoguvacine can reverse tactile allodynia brought on by nerve damage [31]. Large-diameter afferents that are involved in modifying “innocuous sensations” are tightly related to GABA_A_ receptors. The superficial dorsal horn of the spinal cord contains most of these receptors. The evidence points to the critical role of reduced spinal GABA in the initiation and maintenance of neuropathic pain. A continuous or single intrathecal (i.t.) dosage of GABA to the spinal cord or the implantation of GABA-releasing cells reduces the symptoms of neuropathic pain, according to behavioral and pharmaceutical investigations [32,33,34]. On the other hand, inhibiting spinal GABA_A_ receptors worsens the hyperalgesia brought on by peripheral nerve damage [35]. Thus, central sensitization emerges from GABA_A_ receptor blocking at the primary afferent terminals, suggesting the tonic inhibitory role of this receptor in tactile allodynia. Therefore, 6-HF’s strong affinity for GABA_A_ receptors can be used to explain its anti-vulvodynia/allodynic properties. The fact that PTZ can reverse these effects further demonstrates that GABA_A_ receptors are involved in the modulation of neuropathic pain by 6-HF.

The role of opioid receptors in neuropathic pain modulation cannot be overlooked. In a rat model of neuropathic pain, peripherally acting delta opioid receptor agonists have been shown to reduce allodynia [36]. Interestingly, one study demonstrated that in CCI models, the simultaneous activation of mu and delta opioid receptors dramatically reduced neuropathic pain [37]. Opioid receptors are found in the sensory nerves, and they modulate inflammation and wound healing [38]. Endogenous opioid peptides bind to the opioid receptors found on the inflammatory cells in the periphery. As a result, opioidergic medications could reduce pain while also having an anti-inflammatory effect [39,40].

The molecular modeling studies revealed that 6-HF had a considerable affinity for the delta and kappa opioid receptors as well as the GABA_A_ receptor subtypes. Shunting inhibition (increased postsynaptic membrane conductance) and hyperpolarizing inhibition are the two effects on the postsynaptic membrane that are mediated by the ionotropic GABA_A_ receptors (change in membrane potential via the movement of chloride ions). At GABA_A_ receptors, allosteric modulators alter the potency or affinity of agonists such as GABA, hence controlling their action. Due to advancements in our understanding of the roles of GABA_A_ receptor subtypes, these modulators have received a great deal of attention over the past 10 years [27]. Hence, 6-HF exerts a dual effect, i.e., GABA- and opioid-modulating activity, without causing the unwanted side effects.

Research on other synthetic flavonoids, such as 6-hydroxyflavone, 6-methylflavone, 6-methylflavanone, and 6-methoxyflavanone, which are claimed to behave as agonists for GABA_A_ receptor subtypes, has further supported the proposed mechanism [4,41,42]. Given that the importance of the GABAergic and opioidergic systems in these circumstances is well-established [43,44]. Thus, 6-HF post-synaptically activated GABA_A_ receptors, causing chloride ions to traverse across the membrane and the post-synaptic hyperpolarization of the neurons along with a reduction in inflammation.

## 5. Conclusions

6-HF was found to be effective in reducing the pain elicited by DM in the rodent model of neuropathy. The proposed mechanism behind this anti-nociception is the dual effect of 6-HF, that is, the reduction in diabetes-induced inflammation and the allosteric modulation of GABA_A_ and opioid receptors. However, further studies are required to confirm the protective potential of 6-HF in diabetes-induced neuropathy.

The docking studies provided valuable insights into the potential pharmacological effects of 6-HF. It is important to note that computational predictions are not always accurate and must be validated through in vitro and in vivo experiments. In the rat model of neuropathy, 6-HF was found to be beneficial in lowering the pain that was precipitated by DM. The dual action of 6-HF, including a decrease in diabetes-induced inflammation and the allosteric regulation of GABA_A_ and opioid receptors, was the suggested mechanism behind this antinociception. To validate the preventive effect of 6-HF in DIN, however, more research is needed. Our study suggested that flavanones might have potential of treating chronic painful conditions. Further research is needed to determine the specific effects of 6-HF in the context of vulvodynia and to identify the optimal dosing and administration methods for these compounds.

## Figures and Tables

**Table 1:** In vitro COX-2 inhibitory assay: COX-2 enzyme inhibitory assay for the 6-HF and celecoxib analyzed using a COX- 2 (human recombinant) commercial colorimetric COX inhibitor screening assay kit (Cayman test kit-560131; Cayman Chemical Company). *** p < 0.001.

Concentration (µg/mL)	COX-2 % Inhibition (Mean ± S.E.M.)	
	Celecoxib	6-HF
1000	98.13 ± 0.70	82.64 ± 0.75 ***
500	95.17 ± 0.53	77.58 ± 0.77 ***
250	90.39 ± 0.49	74.75 ± 0.63 ***
125	85.13 ± 0.20	68.58 ± 0.70 ***
62.5	81.80 ± 0.37	63.61 ± 0.53 ***
31.25	78.13 ± 0.20	57.79 ± 0.62 ***
IC_50_ (µM)	0.28	21.86

**Table 2:** In vitro 5-LOX inhibitory assay: 5-Lipoxygenase (5-LOX) enzyme inhibitory assay of 6-HF and nordihydroguaiaretic acid (NDGA) as standard using a 5-LOX inhibitory screening kit. *** p < 0.001.

Concentration (µg/mL)	5-LOX % Inhibition (Mean ± S.E.M.)	
	Nor-Dihydro Guaiaretic Acid (NDGA)	6-HF
1000	97.87 ± 0.26	78.37 ± 0.52 ***
500	94.37 ± 1.65	75.90 ± 1.16 ***
250	89.85 ± 0.97	71.48 ± 0.54 ***
125	85.65 ± 1.47	65.56 ± 0.69 ***
62.5	83.17 ± 0.72	61.83 ± 1.07 ***
31.25	71.93 ± 1.13	56.38 ± 0.76 ***
IC_50_ (µM)	0.63	36.00

## Data Availability

Data shall be available on request.
